# Spontaneous ischemic neuropathy of the sciatic nerve due to arterial occlusion – a rare cause of acute neuropathy not to be missed, a report of two cases

**DOI:** 10.1186/s12883-022-02944-3

**Published:** 2022-11-04

**Authors:** Anelia Dietmann, Rascha von Martial, Olivier Scheidegger

**Affiliations:** 1 Department of Neurology, Inselspital, Bern University Hospital, University of Bern, Freiburgstrasse 18, 3010 Bern, Switzerland; 2 Institute of Diagnostic and Interventional Neuroradiology, Inselspital, Bern University Hospital, University of Bern, Bern, Switzerland

**Keywords:** Ischemic neuropathy, Monomelic ischemic neuropathy, Arterial thrombosis, MR neurography, Case report

## Abstract

**Background:**

Ischemic neuropathy of the sciatic nerve without preceding vascular surgical procedures is a rare condition and may be due to arterial occlusion in one limb.

**Case presentations:**

We present two cases with acute onset of pain and sensory symptoms such as pins and needles and numbness in the foot with no or mild motor symptoms. In the neurological work-up, electrophysiological signs of axonal neuropathy of both peroneal and tibial nerves were demonstrated and T2 hyperintensity was seen in the distal sciatic nerves on MR neurography as well as signs indicating arterial thrombosis in the corresponding vessels. Recanalization was obtained in both patients angiographically with significant improvement in one patient.

**Conclusions:**

Spontaneous arterial occlusion of major or peripheral arteries is a rare but important cause of acute onset of single or multiple axonal mononeuropathies of one extremity. Recognition of this infrequent cause is essential since it requires immediate and specific therapeutic options.

## Background

In contrast to polyneuropathies where most or even all peripheral nerves are affected, mostly in a symmetric manner, damage to a peripheral nerve at one site causes focal neuropathies [1]. The most frequent neuropathies of the lower extremity are peroneal neuropathy followed by sciatic neuropathy [2]. Due to its long anatomic course, various sites of sciatic nerve injury are possible with the hip as the most common location of lesions [2]. Sciatic neuropathy can be traumatic, often with more severe clinical presentation depending on the location and severity of injury to the nerve [3]. Iatrogenic sciatic neuropathy due to surgery, most importantly hip replacement surgery, is the most frequent cause of non-traumatic perioperative sciatic neuropathy [4]. In a study of 109 non-traumatic sciatic neuropathy patients, 39 had non-perioperative causes of sciatic neuropathy, most frequently compression (*n* = 16), inflammation (*n* = 8) and idiopathic (*n* = 6). Other less frequent causes were weight loss, infection, radiation, piriformis entrapment, perineuroma and ischemia [4]. In a study on 92 consecutive patients evaluated for sciatic neuropathy, nerve infarction was the third most important cause (10% of patients) after hip arthroplasty and acute external compression [3]. Ischemic sciatic neuropathies have been described in the context of aneurysms with formation of arterial thrombosis on the basis of arteriosclerosis [5], arterial thrombosis after acetabulum fracture and surgery with ilioinguinal approach [6] or intra-operative arterial occlusion during total hip arthroplasty [7]. McManis described six cases of sciatic neuropathy after cardiac surgery. Four of them underwent prolonged periods of intra-aortic balloon pump therapy with a catheter in the femoral artery ipsilateral to sciatic nerve lesion. The other two experienced an ipsilateral femoral artery occlusion [8]. In another case report, ischemic neuropathy of the peroneal and tibial nerve as a rare complication of aorto-femoral bypass surgery has been described [9].

Spontaneous arterial ischemia with secondary axonal nerve damage due to occlusive vascular disease had been first described under the term “ischemic neuritis” in 1949 [10, 11]. Later the term ischemic monomelic neuropathy (IMN) was introduced by Wilburn et al. in 1983 [12] and recently discussed in a report of three cases [13]. Ischemic monomelic neuropathy has been originally described as a non-compressive occlusion of blood supply or steal phenomenon causing single or multiple axonal mononeuropathies in the distal limb, without necrosis of muscles or skin [12]. Causes are typically thromboembolic diseases in cases without preceding vascular surgical procedures [12]. The typical clinical presentation is acute onset of persisting deep, burning pain of a distal extremity, accompanied by paresthesia and numbness, with or without motor weakness [12]. Classical features of limb ischemia such as intermittent ischemic claudication, paleness and swelling of the limb and clinical evidence of necrosis are typically absent and pulses may be palpable [12]. Therefore, a significant and detrimental delay in diagnosis may occur with patients misdiagnosed with lumbar radiculopathy [12] or even complex regional pain syndrome [13].

We present two cases of ischemic sciatic neuropathy due to arterial thrombosis.

## Case presentations

### Patient 1

A 45-year-old male with an unremarkable medical history presented himself to the emergency department with tingling of his right foot during nighttime for two weeks. These symptoms increased to a stabbing pain in his forefoot and pins and needles up to his ankle. Night sleep was markedly disturbed. During daytime, he had no pain but a fluctuating numbness with a decrease in symptoms while moving. Clinical examination revealed numbness of the big toe, medial forefoot and sole, which increased while performing a maximal dorsiflexion of the foot. He had no medication on a regular basis, was an occasional smoker and worked as an electrician. Tarsal tunnel syndrome was postulated based on clinical presentation, and a symptomatic therapy with pregabaline was started.

One week later, he had a follow-up consultation at our center for neuromuscular diseases. Pregabaline (maximum dosage of 150 mg per day) did not show any positive effect. Symptoms were similar, except for a new mild weakness of dorsal extension of the right big toe in the clinical examination. Motor nerve conduction studies (NCS) of the tibial nerve with recording from the right abductor hallucis muscle as well as the peroneal nerve with recording from the extensor digitorum brevis muscle showed significantly reduced amplitudes of the compound muscle action potentials (CMAP), whereas nerve conduction velocity was normal and no nerve conduction block further proximally was detected. The sensory NCS of the right sural nerve was abnormal with reduced conduction velocity and reduced sensory nerve action potentials (SNAP) compared to the left side. Motor and sensory NCS (tibial, peroneal, sural nerve) on the unaffected left side were normal. At this time needle myography of the anterior tibial muscle was normal (Table 1, patient 1). We concluded that there was axonal damage of the tibial and peroneal nerve on the right side or an isolated lesion of the right sciatic nerve. Further laboratory tests were unremarkable including screening for vasculitis, infection or metabolic disease, especially diabetes mellitus.

**Table 1:** Motor and sensory nerve conduction studies of both patients

NCS	Nerve		Right	Left	Normal values (lab/specific)
Patient 1	Peroneal Nerve	CMAP (mV) NCV (m/s)	1.0 38	3.7 45	*2.5* *40*
	Tibial Nerve	CMAP (mV) NCV (m/s)	2.9 44	7.4 60	*5* *37*
	Sural Nerve	SNAP (uV) NCV (m/s)	8 35	22 43	*4* *43*
Patient 2	Peroneal Nerve	CMAP (mV) NCV (m/s)	n.d	0.1 41	*2.5* *40*
	Tibial Nerve	CMAP (mV) NCV (m/s)	n.d	Absent	*5* *37*
	Sural Nerve	SNAP (uV) NCV (m/s)	n.d	3 44	*4* *43*

*NCS* Nerve conduction studies, *CMAP* Compound muscle action potential, *NCV* Nerve conduction velocity, *SNAP* Sensory nerve action potential, *n.d.* Not done

**Fig. 1:**
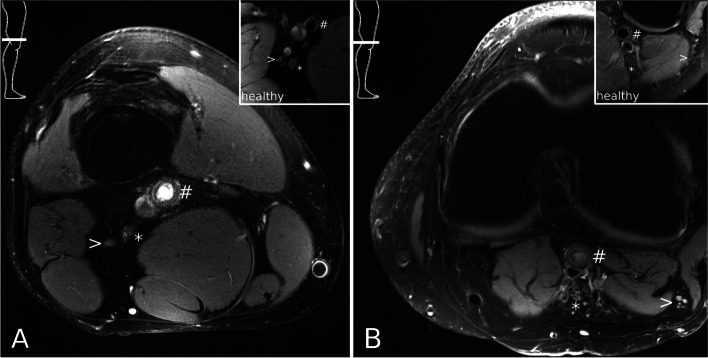
MR Neurography of the popliteal region. Images were acquired at 3 T (Siemens Magnetom SkyraFit, Siemens Healthineers, Erlangen, Germany) using a high resolution, T2-weighted, fat-saturated turbo spin-echo sequence (TR 6000 ms, TE 30 ms, FA 180°, 3 mm slice thickness, FoV 130 mm, matrix size 512). > peroneal nerve, * tibial nerve, # popliteal artery. The smaller images on the upper right part depict the morphology of the nerves and vessels in an age-matched healthy volunteer. **A** Patient 1. Note some hyperintense nerve fascicle bundles in the tibial nerve, and the missing "flow void" of the popliteal artery. **B** Patient 2. Note multiple hyperintense and slightly enlarged nerve fascicle bundles of both tibial and peroneal nerve, and the missing "flow void" of the popliteal artery"

In line with the electrophysiological findings, MR neurography showed a T2 hyperintense signal of the sciatic nerve in the popliteal region on the right side (Fig. 1A). Furthermore, there was a lack of the “flow void” of right femoral and popliteal artery with a concurrent T2 hyperintense perivasal reaction. Sonographically a thrombosis of the right distal femoral artery and popliteal artery was confirmed. The patient underwent an angiography with successful recanalization and percutaneous transluminal angioplasty (PTA) and oral anticoagulation with rivaroxaban and clopidogrel was started.

Three months later electrophysiological examination had completely normalized and angiographic control showed complete revascularization and normal vessel status. The patient had no aneurysm, arteriovenous fistula, cardiac disease or blood coagulation disorder, therefore the cause for arterial thrombosis was idiopathic.

### Patient 2

A 46-year-old male presented himself to an emergency department of a primary care hospital with an acute onset of painful muscle cramps and swelling of his left calf within the last 12 h. At the same time, he noticed numbness in the left big toe and sensation of a colder left foot. He had no motor deficit and no lumbar pain. In the report from the emergency department, a significant swelling of the limb was not described, and a left L5 radiculopathy was suspected. A lumbar MRI was requested showing disc herniation of the lumbar segments L4/5 and L5/S1 with possible compression of nerve roots L5 on the left side as well as S1 on the right side. He was treated with pregabaline, physiotherapy was recommended and a consultation with spinal surgeons was scheduled who later refrained from a surgical intervention.

After one month, he was referred to our center for neuromuscular diseases. He reported that the swelling of the left calf remained with a marked increase in the evenings. Sensations of pins and needles and numbness in the sole of the foot, especially the big toe, reduced slightly after starting pregabaline 300 mg/d. He was a smoker with a history of 40 pack years and consumed eight cannabis joints per day. He noticed slight problems in keeping the balance due to numbness in the sole of the foot.

On examination, again, there was no significant swelling of the calf or foot visible, dorsal flexion of the great toe on the left side was Medical Research Council’s scale (MRC) muscle strength grade M4 with normal strength for all other movements in both feet and legs. He was able to stand and walk on toes and heels. Deep tendon reflexes were brisk and symmetric in all extremities. He was unable to feel light touch, temperature and pinprick in all toes, extending to the forefoot area as well as sole of the foot up to the ankle joint. He complained about allodynia of the dorsal forefoot on light touch.

Motor NCS of the left peroneal nerve with recording on the extensor digitorum brevis muscle revealed a significant reduction of the CMAP amplitude. No motor response was recorded in the NCS of the tibial nerve with recording on the left abductor hallucis muscle (Table 1, patient 2). Sensory NCS showed only mildly reduced sensory amplitudes of the sural nerve. Needle myography showed marked fibrillation potentials and fasciculations in the left anterior tibial muscle, and also mildly in the left lateral head of the gastrocnemic muscle.

All motor and sensory NCS on the unaffected right side were normal (tibial, peroneal, sural nerve). We suspected an axonal lesion either of both peroneal and tibial nerves or of the sciatic nerve. Subsequent MR neurography confirmed an extensive T2 hyperintense neuropathy of the left common and profound peroneal nerve as well as the tibial nerve in the popliteal region (Fig. 1B). Signs of muscle denervation (diffuse T2 hyperintense signal abnormalities) were found in all muscles innervated by the profound peroneal nerve and the tibial nerve. Furthermore, the “flow void” of the popliteal artery was missing indicating arterial thrombosis. Sonographically, arterial thrombosis of the superficial femoral artery including the popliteal artery on the left side was confirmed. A cardio embolic cause of the peripheral arterial embolism was suspected. The patient underwent an angiography with successful recanalization and PTA and oral anticoagulation with rivaroxaban and clopidogrel was started. A four-month follow-up found that paresthesia and pain were only partially reversed and the patient remained on pregabaline. No electrophysiological follow-up examination was available.

## Discussion and conclusions

We present two patients with arterial thrombosis of the distal femoral and popliteal artery with unknown or suspected cardiac source of embolism. Both presented with painful sensory disturbances in one distal leg with only very mild motor deficits. Electrophysiologically, both had signs of axonal sensory-motor neuropathies of the sciatic nerve including peroneal, tibial and sural nerves with low amplitude CMAPs, SNAPs and signs of denervation in the electromyography in the second patient. The secondary ischemic axonal degeneration of the sciatic nerve due to arterial thrombosis was confirmed by MR neurography in both patients.

Unfortunately, the long-term outcome has not been studied sufficiently, especially in the second patient, therefore it is difficult to understand the different outcomes—remission in the first patient after three months and remaining pain and paresthesia in the second patient after four months. A follow-up period of 3–4 months was too short to consider the remaining signs and symptoms in the second patients as a persistent residuum. It would have been important, to follow up the patient after 6 and 12 months, however, the patient preferred to be seen by a treating neurologist close to his place of residence.

The impact of ischemia on peripheral nerves depends on various factors such as site of vessel occlusion, compressive or non-compressive cause of occlusion, acuteness and duration of occlusion and the high anatomical variability of blood supply by nutrient vessels [12]. All nerves receive their blood supply through vasa nervorum that are supplied by nutrient arteries from adjacent vessels in the more distal parts of a limb [11]. The origin and number of the nutrient arteries are highly variable [11]. Studies on animal models demonstrated that in generalised hypoperfusion states such as large vessel occlusion a segmental decreased microcirculation in nerve segments supplied between two adjacent nutrient vessels (“watershed zone”) leads to maximal nerve damage, whereas more distal vascular supply was preserved [14]. Specifically, the popliteal fossa is a developmental watershed due to vascular rearrangements of regression and replacement of the embryonic axial artery in the adjacent thigh and calf, whereas the popliteal artery is a remnant of the embryonic axial artery [15]. This may explain our MR neurography findings of segmental hyperintensities of the nerves in the popliteal region corresponding to focally disrupted blood supply of nutrient arteries with further distal sustained blood supply. 

In conclusion, arterial thrombosis causing ischemic neuropathy may be suspected as a differential diagnosis in patients presenting with acute onset of pain in one limb as the leading symptom together with sensory disturbances being more prominent than motor deficits. Typical clinical signs of limb ischemia may be absent. Besides electrophysiology, MR neurography is a useful tool in localizing the site of the lesion and investigating the cause of acute onset neuropathy. Recognition of arterial thrombosis is essential to immediately initiate revascularization therapy.

## Data Availability

The datasets used and/or analysed during the current study are available from the corresponding author on reasonable request.

## Abbreviations

CMAP: Compound muscle action potential
IMN: Ischemic monomelic neuropathy
MRC: Medical Research Council
MRI: Magnetic resonance imaging
NCS: Motor nerve conduction study
PTA: Percutaneous transluminal angioplasty
SNAP: Sensory nerve action potential
